# Stress-induced microautophagy is coordinated with lysosome biogenesis and regulated by PIKfyve

**DOI:** 10.1091/mbc.E23-08-0332

**Published:** 2024-04-16

**Authors:** Alison D. Klein, Kayla L. Petruzzi, Chan Lee, Michael Overholtzer

**Affiliations:** a BCMB Graduate Program, Weill Cornell Medical College, New York, NY 10065; b Cell Biology Program, Memorial Sloan Kettering Cancer Center, New York, NY 10065; c Gerstner Sloan Kettering Graduate School of Biomedical Sciences, Memorial Sloan Kettering Cancer Center, New York, NY 10065; Children’s Hospital of Philadelphia

## Abstract

Lysosome turnover and biogenesis are induced in response to treatment of cells with agents that cause membrane rupture, but whether other stress conditions engage similar homeostatic mechanisms is not well understood. Recently we described a form of selective turnover of lysosomes that is induced by metabolic stress or by treatment of cells with ionophores or lysosomotropic agents, involving the formation of intraluminal vesicles within intact organelles through microautophagy. Selective turnover involves noncanonical autophagy and the lipidation of LC3 onto lysosomal membranes, as well as the autophagy gene-dependent formation of intraluminal vesicles. Here, we find a form of microautophagy induction that requires activity of the lipid kinase PIKfyve and is associated with the nuclear translocation of TFEB, a known mediator of lysosome biogenesis. We show that LC3 undergoes turnover during this process, and that PIKfyve is required for the formation of intraluminal vesicles and LC3 turnover, but not for LC3 lipidation onto lysosomal membranes, demonstrating that microautophagy is regulated by PIKfyve downstream of noncanonical autophagy. We further show that TFEB activation requires noncanonical autophagy but not PIKfyve, distinguishing the regulation of biogenesis from microautophagy occurring in response to agents that induce lysosomal stress.

## Abstract (executive summary)

Lysosome turnover and biogenesis are coordinated responses to organelle rupture, but whether other forms of lysosomal stress that do not cause rupture engage similar mechanisms is not well understood.

The authors observed that subrupture stress induced by proton ionophores engaged a mechanism of lysosome turnover involving microautophagy, while also activating biogenesis. Microautophagy and biogenesis involved lipidation of the autophagy protein LC3 onto lysosomal membranes, but were distinguished by regulation through the lipid kinase PIKfyve that is required, downstream of LC3, for microautophagy.

These findings identify a coordinated lysosomal stress response that targets lysosomes undergoing stress but not rupture.

## INTRODUCTION

Lysosomal stress responses are numerous and involve signaling mechanisms that either control the biogenesis of new lysosomes, regulated by Transcription Factor EB (TFEB) and related factors (Sardiello *et al.*, 2009), or mediate the repair or turnover lysosomes that have become damaged and undergo rupture (Maejima *et al.*, 2013; Radulovic *et al.*, 2018; Skowyra *et al.*, 2018). Mechanisms of turnover have been largely characterized by studying the responses of cells to agents that induce lysosome rupture, such as the dipeptide L-leucyl-L-leucine methyl ester (LLOMe; Maejima *et al.*, 2013). Whether mechanisms of turnover might also target unruptured lysosomes for elimination in response to stress is not well understood.

Recently, we discovered that the treatment of cells with agents that cause various forms of lysosomal stress, including proton ionophores (Monensin or Nigericin) which raise pH and alter the osmotic potential of the lysosome lumen, induce a form of lysosome turnover that is selective, resulting in the degradation of some, but not all lysosomal transmembrane proteins (Lee *et al.*, 2020). While ruptured lysosomes are known to be sequestered into autophagosomes through a mechanism called “lysophagy” that mediates turnover of whole organelles (Maejima *et al.*, 2013), we found selective turnover instead occurs within intact lysosomes and involves a noncanonical autophagy activity that leads to the formation of intraluminal vesicles (ILVs) from the limiting lysosomal membrane. This stress response involves lipidation of the autophagy protein Microtubule-Associated Protein 1 Light Chain 3 (LC3) directly onto lysosomal membranes, an activity first identified during phagocytosis (Sanjuan *et al.*, 2007) and also referred to as Conjugation of ATG8s onto Single Membranes (CASM; Jacquin *et al.*, 2017; Durgan and Florey, 2022).

Here, we investigate the mechanism of stress-induced micro­autophagy and identify the lipid kinase PIKfyve (Phosphoinositide Kinase, FYVE-Type Zinc Finger Containing) as a regulator of this process. We find that PIKfyve activity is required downstream of non-canonical autophagy for the formation of ILVs, the turnover of LC3 and the lysosomal cation channel Transient Receptor Potential Cation Channel, Mucolipin Subfamily, Member 1 (TRPML1), a previously identified transmembrane target of selective turnover (Lee *et al.*, 2020). We further find that microautophagy is associated with nuclear translocation of the lysosome biogenesis transcription factor TFEB, which occurs in a noncanonical autophagy-dependent, but PIKfyve-independent manner. Together our findings identify PIKfyve as a regulator of microautophagy that acts downstream of noncanonical autophagy, and distinguish selective turnover from biogenesis that occurs in response to treatment with ionophores that induce lysosomal stress but not rupture.

## RESULTS

**FIGURE 1:**
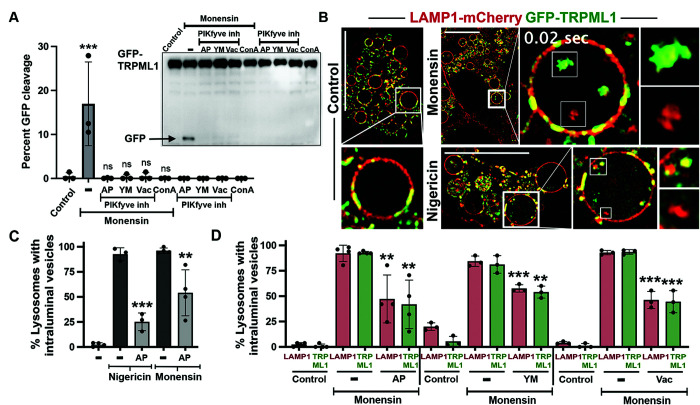
PIKfyve regulates lysosome membrane turnover through microautophagy. (A) GFP cleavage from GFP-TRPML1 induced by treatment with Monensin (lane 2, arrow), a marker of selective lysosome turnover, is inhibited by cotreatment with PIKfyve inhibitors AP, YM, or Vac, as well as the v-ATPase inhibitor ConA. Graph shows mean percent GFP cleavage from GFP-TRPML1 for the indicated conditions, quantified as GFP intensity over GFP-TRPML1 plus GFP, as determined by western blotting. Data from three biological replicates are shown as individual data points and were analyzed using a one-way Anova followed by Dunnett’s test (*p* < 0.0001). Error bars show SD. (B) Images show SIM assay to quantify microautophagy, with Control untreated (left), or Monensin (top right), or Nigericin (bottom right)-treated cells expressing LAMP1-mCherry (red) and GFP-TRPML1 (green). Lysosomes are enlarged for imaging after treatment as described in the *Materials and Methods* section. Note: both LAMP1-mCherry and GFP-TRPML1-positive intraluminal vesicle populations are induced by treatment with Monensin or Nigericin. Insets show enlarged lysosomes either with (right images) or without (left control image) ILVs; see also Supplemental Video 1. Scale bar = 10 μm. (C) Graph shows quantification of microautophagy in response to Nigericin or Monensin in the presence or absence of AP, shown as percent lysosomes positive for ILVs from at least three independent biological replicates, represented by individual data points. *n* > 45 lysosomes per replicate; error bars show SD. A one-way Anova followed by Dunnett’s test was performed (from left to right; ***p < 0.0001, **p = 0.003). (D) Graph shows percent lysosomes with either LAMP1-mCherry or GFP-TRPML1-positive ILVs in cells treated with Monensin in the presence or absence of the PIKfyve inhibitors AP, YM, or Vac. *n* > 45 lysosomes per replicate. PIKfyve-inhibited conditions were compared with Monensin controls by one-way Anova followed by Dunnett’s test; from left to right **p = 0.0032, **p = 0.0011, ***p = 0.0005, **p = 0.0044, ***p < 0.0001, ***p = 0.0002.

To investigate if the lysosomal lipid kinase PIKfyve, which catalyzes the formation of phosphatidylinositol (5)-phosphate (PI(5)P) and phosphatidylinositol (3,5)-bisphosphate (PI(3,5)P_2_) on late endosomal and lysosomal membranes, might regulate stress-induced microautophagy, cells were treated with the sodium-proton ionophore Monensin and turnover of the lysosomal cation channel TRPML1 was examined through a GFP cleavage assay that we previously reported (Lee *et al.*, 2020). As shown in Figure 1A, treatment with any of three different PIKfyve inhibitors, Apilimod (AP; Cai *et al.*, 2013), YM201636 (YM; Jefferies *et al.*, 2008), or Vacuolin-1 (Vac; Sano *et al.*, 2016; Kang *et al.*, 2020), significantly reduced the cleavage of GFP from GFP-TRPML1, similar to treatment with the v-ATPase inhibitor Concanamycin A (ConA), demonstrating that PIKfyve activity is required for lysosomal membrane protein turnover through this mechanism.

To further examine whether PIKfyve activity is required for microautophagy, an imaging-based approach was developed to quantify the formation of ILVs using structured illumination microscopy (SIM) (Figure 1B; Supplemental Video 1). Like the effect on GFP-TRPML1 turnover, the inhibition of PIKfyve activity significantly reduced the formation of ILVs in response to treatment with Monensin or the potassium-proton ionophore Nigericin, another inducer of selective turnover (Lee *et al.*, 2020), demonstrating a requirement of PIKfyve for ILV formation through microautophagy (Figure 1C). Interestingly, while the LAMP1 lysosomal transmembrane protein (Lysosome-Associated Membrane Protein 1) was shown previously to be excluded from this form of selective turnover (Lee *et al.*, 2020), an expressed tagged LAMP1-mCherry protein was observed to localize to ILVs at the same rate as GFP-TRPML1, and in a PIKfyve-regulated manner (Figure 1D).

**Figure:** Movie S1 **Structured Illumination Microscopy (SIM) imaging of intraluminal vesicles**. Images show cells expressing LAMP1‐mCherry (red) and GFP‐TRPML1 (green) treated with Monensin to induce microautophagy and then Apilimod to enlarge lysosomes for live cell SIM imaging at 20 milli‐second intervals; see also Figure 1B. Boxed region shows enlarged region on right with time‐lapse imaging of ILVs. Note both LAMP1‐mCherry and GFP‐TRPML1 are localized to intraluminal vesicles that move rapidly within the lysosome lumen. Scale bar included in Figure 1B.

**FIGURE 2:**
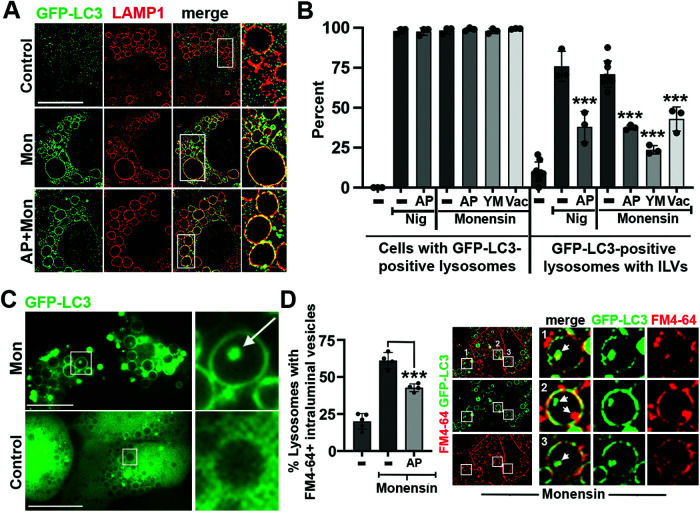
Imaging approaches demonstrate PIKfyve regulates microautophagy downstream of LC3 lipidation. (A) Images show immunofluorescence staining of GFP-LC3 (green) and LAMP1 (red) in control (top), Monensin (middle), or Monensin +AP (bottom)-treated cells. Boxed regions are enlarged in the right panels. Scale bar = 10 μm. (B) Graphs show quantification of GFP-LC3 lipidation and GFP-LC3-positive intraluminal vesicle populations through live imaging analysis. Percent cells positive for GFP-LC3-labeled lysosomes (left; *n* = 100 cells per replicate), and percent lysosomes positive for ILVs (right; *n* > 45 lysosomes per replicate) from at least three independent biological replicates are shown, represented by individual data points; error bars show SD. Data were analyzed using a one-way Anova followed by Dunnett’s test. *P* values comparing ionophores Nigericin or Monensin to the indicated inhibitors listed in order of conditions from left to right: ***p < 0.0001, *p* < 0.0001, *p* < 0.0001, *p* < 0.0001. (C) Images show representative live imaging of GFP-LC3-expressing *sgATG13* cells treated with Monensin (top) or under control conditions (bottom). Boxed regions are enlarged on right; arrow indicates intraluminal vesicle. Scale bars = 10 μm. See also Supplemental Video 2. (D) Images show cells expressing GFP-LC3 (green) incubated with membrane dye FM4-64 (red) and treated with Monensin to induce microautophagy. Graphs show quantification of GFP-LC3 and FM4-64-positive intraluminal vesicle populations through live imaging analysis. Percent lysosomes positive for ILVs (*n* > 10 cells per replicate) from at least three independent biological replicates are shown, represented by individual data points; error bars show SD; ***p = 0.0005; one-way Anova followed by Dunnett’s test.

Stress-induced microautophagy was previously shown to be associated with lipidation of the autophagy protein LC3 onto lysosomal membranes and to require the autophagy gene *ATG5,* whose product directs LC3 lipidation when conjugated to Atg12 and in complex with Atg16L, but not *ATG13,* which encodes part of a nutrient-regulated signaling complex that functions upstream in canonical autophagy (Florey and Overholtzer, 2012; Lee *et al.*, 2020). Two methods were used to investigate whether PIKfyve might be required for microautophagy upstream or downstream of LC3 lipidation. First, GFP-LC3-expressing *sgATG13* cells, which are defective for macroautophagy but competent for lysosomal LC3 lipidation (Lee *et al.*, 2020), were treated with Monensin in the presence or absence of PIKfyve inhibitors (Figure 2), and second, PIKfyve expression was knocked-down by siRNA (Supplemental Figure 1A); both methods were used to examine LC3 localization by SIM. As shown in Figure 2A, Monensin treatment induced large ring-like structures of GFP-LC3 throughout the cytoplasm that were colocalized with LAMP1, consistent with lipidation on enlarged lysosomes, as reported (Florey *et al.*, 2015; Jacquin *et al.*, 2017). Inhibition of PIKfyve did not reduce the Monensin-induced localization of GFP-LC3, suggesting that LC3 lysosomal lipidation might occur in a PIKfyve-independent manner (Figure 2, A and B; Supplemental Figure 1B). Consistent with this, immunostaining of endogenous LC3 also showed a high degree of lysosomal lipidation in response to Monensin or Nigericin treatment that was not inhibited by treatment with AP (Supplemental Figure 2A). In addition to lysosomal LC3 lipidation, we noted in live cell imaging the presence of many ILVs with GFP-LC3, which were identified, like those formed with lysosomal membrane proteins, by their intraluminal localization and rapid movement (Figure 2C; Supplemental Video 2). While PIKfyve inhibition with any of three inhibitors or siRNAs did not affect the lipidation of GFP-LC3 onto lysosomes in response to Monensin or Nigericin, it significantly reduced the appearance of GFP-LC3 on ILVs, consistent with a requirement of PIKfyve for microautophagy occurring downstream of LC3 lipidation onto lysosomal membranes (Figure 2B; Supplemental Figure 1B). We further examined ILV formation in GFP-LC3-expressing *sgATG13* cells by labeling endosomes with the fluorescent membrane dye FM 4-64, which also allowed for visualization of ILVs induced by Monensin treatment, independent of lysosomal markers and in a cell line that is deficient for canonical autophagy (Florey *et al.*, 2015; Jacquin *et al.*, 2017; Lee *et al.*, 2020). As shown in Figure 2D, the inhibition of PIKfyve with AP reduced the frequency of ILVs labeled with FM 4-64, demonstrating, again, that Monensin-induced ILV formation is regulated by PIKfyve.

**Figure:** Movie S2 **Time‐lapse imaging of GFP‐LC3 lipidation and intraluminal vesicles**. Images show *sgATG13* cells expressing GFP‐LC3 treated with Monensin to induce microautophagy and then Apilimod to enlarge lysosomes for live cell imaging at 20 milli‐second intervals; see also Figure 2C. Note prominent cytoplasmic rings corresponding to enlarged lysosomes are visible with ILVs noted by their intraluminal localization and rapid movement. Left image shows large area with two individual cells; right images show additional examples. Scale bar on left image = 10μm; scale bars on right = 2μm.

**FIGURE 3:**
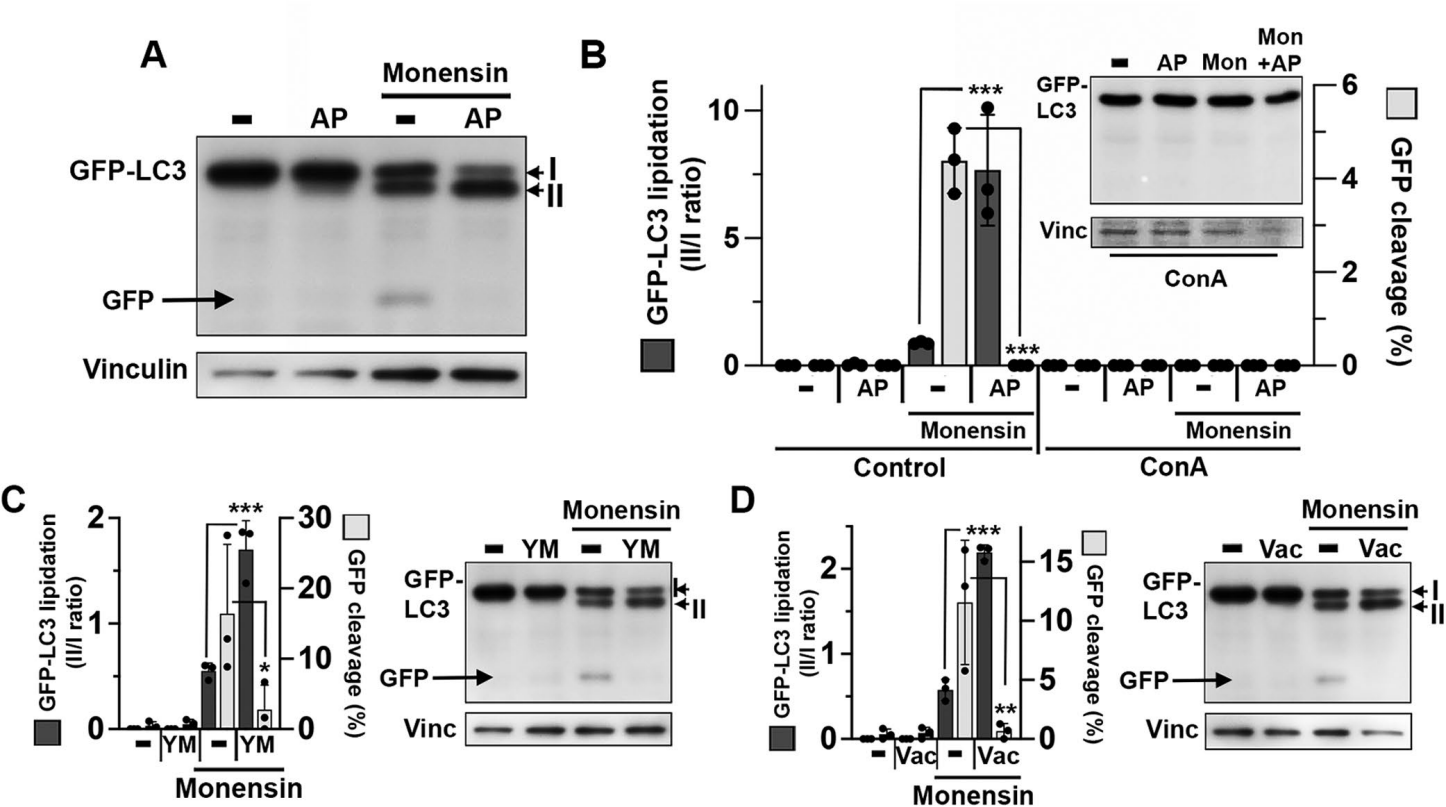
PIKfyve regulates microautophagy downstream of LC3 lipidation by western botting. (A) Western blot shows GFP-LC3 lipidation and GFP cleavage in response to treatment with Monensin in the presence or absence of AP in *sgATG13* cells. Note GFP-LC3 lipidation (indicated as form II vs. I) and GFP cleavage are induced by Monensin treatment. AP cotreatment inhibits GFP cleavage and leads to enhanced lipidation. (B) Graph shows quantification of GFP-LC3 lipidation (left axis, dark gray bars) and GFP cleavage (right axis, light gray bars). GFP-LC3 lipidation was quantified as II/I ratios; GFP cleavage as percent GFP over total GFP-LC3 I and II plus GFP. Data are means from three independent experiments shown as individual data points; error bars indicate SD. Data were analyzed using a one-way Anova followed by Dunnett’s test; note: Monensin-induced GFP cleavage is significantly reduced by AP (***p < 0.0001), while Monensin-induced GFP-LC3 lipidation is enhanced by AP (***p < 0.0001). Inset shows western blotting from cells treated with ConA, quantified on the right side of the graph. (C) Graphs show quantification of GFP-LC3 lipidation (left axis, dark gray bars) and GFP cleavage (right axis, light gray bars), as in part B, for cells treated with Monensin in the presence or absence of YM. Data were analyzed using a one-way Anova followed by Dunnett’s test (from left to right: ***p = <0.0001, *p = 0.0320. (D) Graphs show quantification of GFP-LC3 lipidation (left axis, dark gray bars) and GFP cleavage (right axis, light gray bars), as in part E and F, for cells treated with Monensin in the presence or absence of Vac and was analyzed using a one-way Anova followed by Dunnett’s test (from left to right: ***p < 0.0001, **p = 0.0027). Representative western blots for C and D are shown as in part A.

To further examine the relationship between PIKfyve activity and noncanonical autophagy, cell lysates were examined for LC3 lipidation and turnover. Treatment with Monensin induced the lipidation of GFP-LC3 in *sgATG13* cells (Figure 3A) in a manner dependent on v-ATPase activity (Figure 3B), which is another defining feature of noncanonical autophagy that targets lysosomal membranes (Florey *et al.*, 2015; Jacquin *et al.*, 2017). In addition to lipidation, we observed that GFP was cleaved from GFP-LC3 in a v-ATPase and cathepsin protease-dependent manner, consistent with lysosomal turnover that would be expected due to its localization on ILVs (Figure 3, A and B; Supplemental Figure 2B). The inhibition of PIKfyve by treatment with AP did not inhibit, but instead enhanced the extent of GFP-LC3 lipidation, and also blocked the appearance of cleaved GFP (Figure 3, A and B), demonstrating a requirement of PIKfyve for the formation of ILVs but not for LC3 lipidation that occurs upstream. Like AP treatment, the inhibition of PIKfyve with YM or Vac also reduced GFP cleavage and led to enhanced GFP-LC3 lipidation in Monensin-treated cells (Figure 3, C and D). The enhanced GFP-LC3 lipidation observed in PIKfyve-inhibited cells may be due to a lack of turnover that requires the formation of ILVs.

To investigate whether TRPML1, a known effector of PIKFyve (Dong *et al.*, 2010), might also be involved in microautophagy, cells were treated with pharmacological agents that affect TRPML1 activity and the effects on ILV formation and GFP-LC3 turnover were examined. Treatment with the TRPML1 activating compound ML-SA1 (Shen *et al.*, 2012), which has been previously shown to induce the lysosomal lipidation of LC3 (Goodwin *et al.*, 2021) induced the formation of ILVs with LAMP1-mCherry and GFP-TRPML1, similar to Monensin and Nigericin (Supplemental Figure 2C). In Monensin-treated cells, the inhibition of TRPML1 by treatment with inhibitors ML-SI1 and ML-SI3 reduced ILV formation (Supplemental Figure 2D), as well as GFP-LC3 turnover (Supplemental Figure 2E), consistent with TRPML1 regulating this process (Shen *et al.*, 2012; Samie *et al.*, 2013). TRPML1 inhibition did not reduce GFP-LC3 lipidation, but instead enhanced it (Supplemental Figure 2E), suggesting that TRPML1, like PIKfyve, is required for ILV formation but not LC3 lipidation in ionophore-treated cells.

**FIGURE 4:**
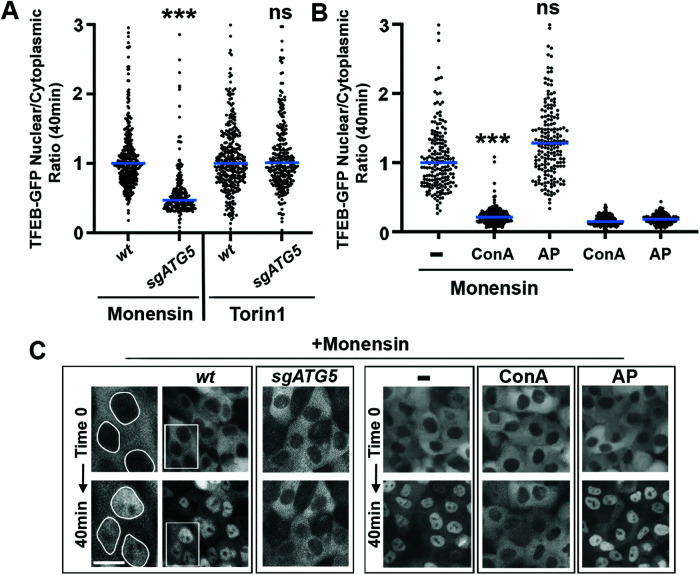
Microautophagy occurs with PIKfyve-independent activation of TFEB. (A) Graph shows fold nuclear localization of TFEB-GFP in response to treatment with Monensin or Torin1, in *wt* or *sgATG5* cells. Note *ATG5* regulates the induction of nuclear TFEB-GFP in response to Monensin but not Torin1. Data show quantification of nuclear to cytoplasmic ratios from cells examined by time-lapse microscopy from three independent biological replicates, with *n* > 75 per replicate. Each cell is represented by an individual data point; bars show medians. Data are normalized to the median nuclear induction in *wt* cells. *P* values comparing treatment of Monensin or Torin1, in *wt* to *sgATG5* cells; *p* = 0.0009; ns, *p* = 0.1304 (Unpaired *t* test). See Supplemental Video 3. (B) Nuclear translocation of TFEB-GFP is inhibited by treatment with the v-ATPase inhibitor ConA but not the PIKfyve inhibitor AP. Data show quantification of nuclear to cytoplasmic ratios from cells examined by time-lapse microscopy from three independent biological replicates, with *n* > 75 per replicate. Each cell is represented by an individual data point; bars show medians. Data are normalized to the median nuclear induction by Monensin.***p < 0.0001 (Unpaired *t* test). See Supplemental Video 4. (C) Images show representative fields of view from time-lapse imaging for the indicated conditions quantified in parts A and B; see also Supplemental Videos 3 and 4. Left images show insets as enlarged regions with nuclei encircled in white. Scale bar = 10 μm.

**FIGURE 5:**
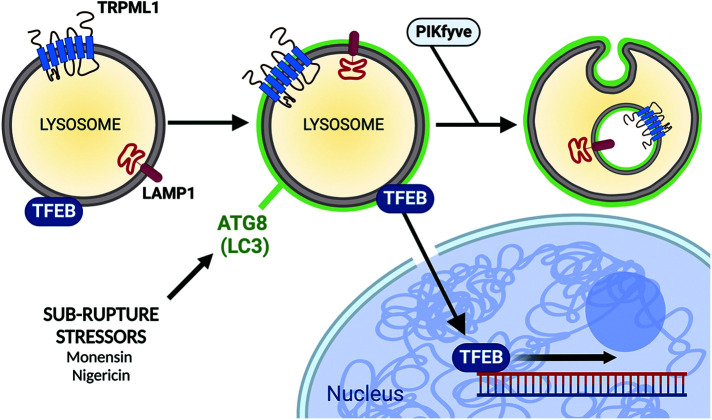
Model: selective lysosome membrane turnover through stress-induced microautophagy involves the *ATG5*- and v-ATPase-dependent lipidation of LC3 onto lysosomal membranes that do not undergo rupture. LC3 and transmembrane proteins localize to ILVs and subsequently undergo turnover. The activity of PIKfyve is required for efficient formation of ILVs and for the turnover of TRPML1 and LC3, but not for LC3 lipidation onto lysosomal membranes that occurs upstream. Like microautophagy, the transcription factor TFEB is activated in an *ATG5*- and v-ATPase-dependent manner, consistent with its induction by noncanonical autophagy. However, TFEB activation occurs independently of PIKfyve, consistent with the model that PIKfyve supports the formation of ILVs during microautophagy but is not required for LC3 lipidation that occurs upstream. Created with Biorender.com.

It was recently reported that the targeting of lysosomal membranes by noncanonical autophagy can induce the nuclear translocation of TFEB, a transcription factor that upregulates a gene expression program that drives lysosome biogenesis (Lang *et al.*, 1998; Goodwin *et al.*, 2021). To examine whether TFEB might be activated in the context of selective turnover, control and *ATG5* knockout cells expressing TFEB-GFP were treated with Monensin and nuclear versus cytoplasmic localizations were quantified by time-lapse microscopy. Treatment with Monensin resulted in the rapid translocation of TFEB-GFP into the nucleus, in an *ATG5*-dependent, and v-ATPase-dependent manner, consistent with the activation of lysosome biogenesis occurring as a result of noncanonical autophagy, as reported (Figure 4; see Supplemental Videos 3 and 4; Florey *et al.*, 2015; Jacquin *et al.*, 2017; Goodwin *et al.*, 2021). To examine whether PIKfyve regulates the activation of TFEB, cells were treated with Monensin in the presence or absence of AP. As shown in Figure 4B and Supplementary Video 4, the inhibition of PIKfyve did not inhibit the rapid nuclear translocation of TFEB-GFP induced by Monensin, demonstrating that stress-induced microautophagy and lysosome biogenesis both occur downstream of noncanonical autophagy but are regulated through separable mechanisms (Figure 5).

**Figure:** Movie S3 **TFEB‐GFP activation by Monensin is regulated by ATG5**. Time‐lapse images show representative wt and *sgATG5* cells expressing TFEB‐GFP treated as indicated and used for quantification of TFEB‐GFP nuclear localization in Figure 3A; see also images in Figure 3C. Note *ATG5* is required for TFEB‐GFP nuclear localization in response to Monensin treatment but not Torin1. Scale bar included in Figure 3C.

**Figure:** Movie S4 **TFEB‐GFP activation by Monensin requires v‐ATPase activity but not PIKfyve**. Time‐lapse images show representative cells expressing TFEB‐GFP treated as indicated and used for quantification of TFEB‐GFP nuclear localization in Figure 3B; see also images in Figure 3C. Note ConA treatment inhibits nuclear localization in induced by Monensin but Apilimod has no effect. Scale bar included in Figure 3C.

## DISCUSSION

Here we investigate stress-induced microautophagy and identify the lipid kinase PIKfyve as a regulator of this process. We find that PIKfyve is required for the formation of ILVs generated in response to treatment with stressors that induce the lipidation of LC3 onto lysosomal membranes. We further find that the lysosome biogenesis transcription factor TFEB is translocated to the nucleus during selective turnover, in an autophagy gene-dependent, but PIKfyve-independent manner. Thus, while selective lysosome turnover and biogenesis pathways are induced together in response to noncanonical autophagy, these opposing processes are distinguishable with respect to regulation by PIKfyve.

The mechanism underlying how PIKfyve regulates microautophagy remains to be further explored. In yeast, activity of the PIKfyve ortholog Fab1 has been shown to regulate microautophagy through a mechanism involving either the trafficking of proteins that form raft-like domains in the vacuole membrane (Tsuji *et al.*, 2017), or through its well established function in controlling vacuole fission, a function shared by PIKfyve that is required for lysosome fission in mammalian cells (Krishna *et al.*, 2016; Choy *et al.*, 2018). Fission could support microautophagy indirectly by generating a pool of lysosomes with properties suitable for the formation of ILVs (Takuma and Ushimaru, 2022). The function of TRPML1 in regulating fission (Dong *et al.*, 2010; Krishna *et al.*, 2016; Cao *et al.*, 2017), and the requirement shown here for TRPML1 to support microautophagy could conceivably link these processes, although further studies are needed to investigate the role of TRPML1 beyond the pharmacological approaches that we have utilized.

While TRPML1 activation is reported to be sufficient to activate lysosomal LC3 lipidation, our findings clearly identify PIKfyve and TRPML1 as functioning downstream of LC3 lipidation in ionophore-treated cells. LC3 can interact with key factors that promote the formation of ILVs, including the neutral sphingomyelinase 2 (nSMase2)-activating protein, Factor Associated with nSMase2 activity (FAN), a regulator of ILV formation linked to the formation of ceramide-enriched microdomains in the lysosomal membrane (Leidal *et al.*, 2020), and also ESCRT proteins (Katzmann *et al.*, 2001). ESCRT-III was recently shown to recruit to lysosomes treated with Monensin or Nigericin and to be required for the induction of microautophagy (Babst *et al.*, 2002; Ogura *et al.*, 2023). One particular subset of LC3-related proteins, including GABARAP, GABARAPL1, and GABARAPL2, three orthologues of a larger LC3-related family of six proteins with homology to yeast ATG8 (Ichimura *et al.*, 2000; Wesch *et al.*, 2020), recruit ESCRT-III, potentially through specific interaction with the adaptor protein ALIX (Ogura *et al.*, 2023). As ESCRT-III deforms membranes in support of the formation of ILVs (Hanson *et al.*, 2008), interaction with PIKfyve and TRPML1 activity in this context may be interesting to investigate. TRPML1 itself has also been shown to bind to LC3 (Nakamura *et al.*, 2020), and further studies may explore the intriguing possibility that TRPML1 is selected for turnover by this mechanism.

Lysosome-targeted LC3 lipidation was recently shown to induce the nuclear translocation of TFEB, consistent with our findings here (Nakamura *et al.*, 2020), and this was also demonstrated to require the same subset of LC3-related proteins, named GABARAP (Goodwin *et al.*, 2021). The GABARAP-dependent recruitment of a complex called FLCN/FNIP to lysosomal membranes resulted in the nuclear translocation of TFEB due to inhibition of phosphorylation mediated by mechanistic target of rapamycin complex 1 (mTORC1; Meng and Ferguson, 2018; Goodwin *et al.*, 2021). Interestingly, PIKfyve activity has also been shown to regulate TFEB by supporting its interaction with mTORC1 (Hasegawa *et al.*, 2022). Here, we find that the rapid activation of TFEB induced by lysosomal stress does not require PIKfyve activity, suggesting, overall, that LC3-related proteins may coordinate biogenesis and turnover in response to stress by engaging FLCN/FNIP-dependent activation of TFEB, while activating PIKfyve-dependent microautophagy. Further studies may reveal whether PIKfyve-independent regulation of TFEB in ionophore-treated cells involves TRPML1.

It remains to be explored why lysosomes undergo this selective form of turnover in response to agents that induce lysosome stress but do not cause rupture (see Supplemental Video 5; Lee *et al.*, 2020; Ogura *et al.*, 2023). Recent findings in senescent cells and in *C. elegans* suggest that stress-induced microautophagy could actually inhibit lysosomes from rupturing (Ogura *et al.*, 2023). It is conceivable that microautophagy and turnover of selected lysosomal proteins could relieve stress on the membrane that is due to swelling or membrane stretch (Florey *et al.*, 2015), or could sequester damaged regions of the limiting membrane onto ILVs (Ogura *et al.*, 2023). Microautophagy could also be involved maintaining lysosome size or degradative capacity (Lee *et al.*, 2020). Unbiased approaches to define lysosome composition could further define the extent of remodeling that occurs and uncover how stress-induced microautophagy affects lysosome function. While we have shown that selected transmembrane proteins undergo turnover (Lee *et al.*, 2020), our current study shows proteins that undergo turnover (TRPML1) and those that do not (LAMP1) are both found on ILVs, suggesting that turnover requires but is not determined by vesicle formation. Further studies investigating the composition and fate of ILVs may reveal how particular transmembrane proteins are targeted for degradation. Finally, our study reveals that LC3 is localized to ILVs and undergoes degradation as a result of this mechanism. While LC3 flux is a known hallmark of canonical autophagy, our findings reveal that LC3 flux can also be a feature of noncanonical autophagy, through the formation and turnover of ILVs derived from the limiting lysosomal membrane.

**Figure:** Movie S5 **Monensin or Nigericin treatment do not induce lysosome rupture**. Time‐lapse images show cells expressing GFP‐Gal3 and treated as indicated. Note LLOMe treatment induces the rapid appearance of GFP‐Gal3 puncta due to lysosome rupture but Monensin and Nigericin treatment do not.

## MATERIALS AND METHODS

### Cell culture and reagents

MCF10A cells (American Type Culture Collection [ATCC]) were cultured as described (Lee *et al.*, 2020), in DMEM/F12 with 5% heat-inactivated horse serum, plus EGF (Peprotech), insulin (Sigma), cholera toxin (Sigma), hydrocortisone (Sigma), and pen/strep at 37°C in 5% CO_2_. MCF10A *sgATG5* and *sgATG13* cells, and cells expressing GFP-Galectin3 (GFP-Gal3), GFP-LC3, LAMP1-mCherry, and GFP-TRPML1 were previously described (Florey *et al.*, 2011; Lee *et al.*, 2020). Cell lines were tested for mycoplasma using Dapi staining or Mycoalert detection kit (Lonza; Catalogue#LT07-218) bimonthly. For ILV quantification, a clonal population was used to normalize LAMP1-mCherry and GFP-TRPML1 expression between cells. Cells expressing TFEB-GFP were generated by retroviral transduction as described (Lee *et al.*, 2020). The following reagents were used: L-leucyl-L-leucine methyl ester (LLOMe; 0.5μM; Cayman Chemicals; Catalogue# 16008-100), Monensin (50 μM; Sigma-Aldrich; Catalogue#M5273-1G), Nigericin (25 μM; Tocris Bioscience; Catalogue# 43-1210), AP (200 nM; [Axon Medchem]), YM (1 μM; SelleckChem; Catalogue# S1219-10MM/1 ML), Vac (1 μM; EMD Millipore; Catalogue# 673000-10MG), Torin1 (0.5 µM; Tocris Bioscience; Catalogue# 42-471-0), and ConA; 100 nM; Sigma-Aldrich; Catalogue# C9705-.1MG). ML-SA1 (20 μM; MedChem Express; Catalogue# HY-108462), ML-SI1(25μM or 50 μM; MedChem Express; Catalogue# HY-134818), ML-SI3 (10 μM; Selleck Chem; Catalogue# E0026). Pepstatin A (10 μg/ml; Tocris Bioscience; Catalogue# 1190), and E64d (10 μg/ml; Tocris Bioscience; Catalogue# 4545).

### Western blotting

Cells were plated at 350,000 per sixwell for the indicated treatments and then harvested in lysis buffer (50 mM Tris Cl pH 7.5, 150 mM NaCl, 1 mM EDTA pH 8.0, 2% sodium dodecyl sulfate), boiled at 100°C for 5 min, and vigorously pipetted to remove viscosity. Note, PIKfyve inhibitors were added 30 min before the addition of Monensin, which was added for 3.5 h followed by a 1-h chase in the presence or absence of PIKfyve inhibitors. Protein concentrations were measured by BCA assay (Thermo Fisher Scientific) and western blotting was performed as described (Lee *et al.*, 2020). Blots were analyzed using the Amersham Imager 600 (GE Healthcare) and quantifications were performed on unsaturated images using ImageJ. The following primary antibodies were used: anti-GFP (1:400; Catalogue#2555, Cell Signaling Technology [CST]) and anti-Vinculin (1:400; CST; Catalogue# 4650S).

### Time-lapse widefield microscopy

Time-lapse epifluorescence imaging was performed using a Nikon Ti-E microscope enclosed in an environmental chamber maintained at 37°C and 5% CO_2_. Cells expressing GFP-Gal3 or TFEB-GFP were cultured on 35 mm glass-bottomed dishes (MatTek), treated as indicated, and immediately mounted onto the microscope for image acquisition at 10 min intervals. For TFEB-GFP, changes in nuclear:cytoplasmic intensity were quantified using ImageJ and calculated as ratios after 40 min over time 0, using background subtraction and normalization to the median change in Monensin in control or *wt* cells, as indicated.

### Elyra 7 Live Cell Imaging and SIM

Quantification of ILVs and GFP-LC3-localization to lysosomes was carried out using the Elyra 7 super-resolution microscope and Zen Pro software (Zeiss). One day before treatment, 200,000 cells were plated on 35-mm glass-bottomed dishes, and cells were then treated in the presence or absence of PIKfyve inhibitors (AP, YM, or Vac) for 30min, followed by an additional 2-h incubation with Monensin or Nigericin. To allow for visualization of ILVs, cells under all conditions were treated with PIKfyve inhibitors (AP, YM, or Vac, as indicated) at the end point of the assay to enlarge lysosomes. Percent lysosomes positive for LAMP1-mCherry and/ or GFP-TRPML1-labeled ILVs were quantified as indicated. GFP-LC3 localization to lysosomes was quantified as percent cells with at least five large GFP-LC3-positive ring structures (greater than or equal to 2 μm diameter) observed in the cytoplasm.

### Immunocytochemistry

Cells were seeded on 35-mm glass-bottomed dishes (MatTek) 1 d before treatment in the presence or absence of AP for 30min, followed by Monensin for 1 h. To allow for visualization of GFP-LC3 lipidation onto lysosome membranes, cells under all conditions were treated with AP at the end point of the assay to enlarge lysosomes. Cells were fixed using ice cold 1:1 Methanol/Acetone for 15 min at –20°C, followed by three 5-min phosphate-buffered saline (PBS) washes, and overnight incubation with primary antibodies at 4°C. Samples were then washed three times with PBS for 5min each and then incubated with secondary antibodies for 1 h at room temperature, followed by three 5-min PBS washes. The following antibodies were used: anti-LAMP1 (1:500; BD Biosciences; catalogue# 555798), anti-LC3B (1:500; Abcam; catalogue# EPR18709), and Alexa Fluor 568 and 488 goat antimouse and antirabbit secondaries (1:500; Life Technologies). Images were acquired using the Elyra 7 super-resolution microscope and analyzed by SIM using ZenPro software.

### PIKfyve siRNA

Sixty-thousand cells were plated overnight in sixwell dish and transfected with Lipofectamine RNAiMAX (Invitrogen) and 100 nM of siRNA (Dharmacon) in OPTI-MEM+ GlutaMax (Life Technologies). After 16 h, cells were washed and refed with growth medium. For western blotting, cells were lysed after 48 h. For imaging, cells were plated 24 h after refeeding onto glass bottom dishes (MatTek) and imaged using the Elyra 7 super-resolution microscope and analyzed by SIM. The following siRNAs were used: siGENOME Non-Targeting siRNA Pool #2 (Catalogue# D-001206-14-20); siGENOME Human PIKFYVE (200576) siRNA (Catalogue# D-005058-09-0010) Target Sequence: GAAUGGAGUUUCAGGAUCA (siRNA #1); siGENOME Human PIKFYVE (200576) siRNA (Catalogue#D-005058-10-0010) Target Sequence: GGAAAUCUCCUGCUCGAAA (siRNA #2).

### Statistical analysis

Statistical analysis was performed using GraphPad Prism 9. The number of biological replicates is three unless otherwise stated. The statistical analysis performed and post hoc tests used are mentioned in figure legends where statistical significance is denoted by ∗∗∗, *p* < 0.001; ∗∗, *p* < 0.01; or ∗, *p* < 0.05; ns, not significant.

## Supplementary Material



## Abbreviations used:

ALIX: ALG-2-interacting protein X
AP: Apilimod
ATG: autophagy-related
CASM: conjugation of ATG8s onto single membranes
ConA: Concanamycin A
ESCRT: endosomal sorting complex required for transport
FAN: factor associated with nSMase2 activity
FLCN: folliculin
FNIP: folliculin interacting protein 1
GABARAP: GABA(A) receptor associated protein
GFP: green fluorescent protein
ILV: intraluminal vesicle
LAMP1: lysosome-associated membrane protein 1
LC3: microtubule-associated protein 1 light chain 3
LLOMe: L-leucyl-L-leucine methyl ester
nSMase2: neutral sphingomyelinase 2
PIKfyve: phosphoinositide kinase FYVE-type zinc finger containing
TFEB: transcription factor EB
TRPML1: transient receptor potential cation channel, mucolipin subfamily, member 1
Vac: vacuolin-1
v-ATPase: vacuolar-type ATPase
YM: YM201636
