# Supplementary material for: Stress-induced microautophagy is coordinated with lysosome biogenesis and regulated by PIKfyve
Source: Mol Biol Cell. 2024 Apr 16;35(5):ar70. doi: 10.1091/mbc.E23-08-0332 (PMC11151102; doi:10.1091/mbc.E23-08-0332)
Supplement: Supplementary file 6 [file mbc-35-ar70-s001.pdf]

Supplemental Materials

Molecular Biology of the Cell

Klein *et al*.

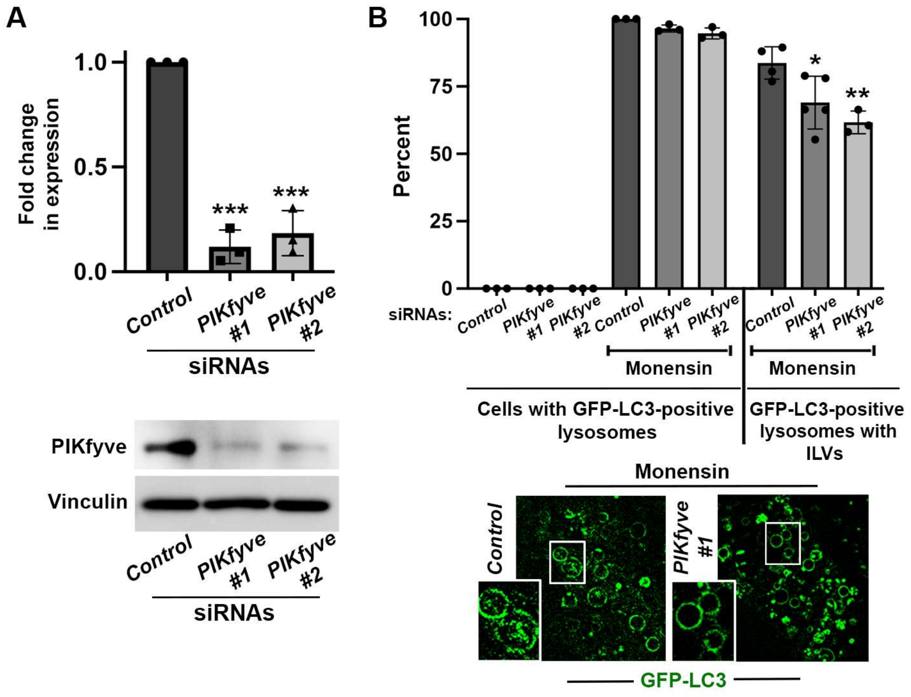

Supplementary Figure 1

Supplementary Figure 1: siRNA mediated knockdowns of PIKfyve inhibit vesicle formation but not LC3 lipidation onto lysosomal membranes. (A) Graph shows fold change in PIKfyve expression normalized to Vinculin loading control in siRNA control and *PIKfyve*-treated cells. Representative Western blot shows reduction in PIKfyve protein expression compared to control. *PIKfyve* siRNAs #1 and #2 both were compared to siRNA control with one-way Anova followed by Dunnett's test, ***p<0.0001 for each. (B) Graph shows quantification of GFP-LC3 lipidation and GFP-LC3-positive intraluminal vesicle populations through live imaging analysis. Percent cells positive for GFP-LC3-labeled lysosomes (left; n=100 cells per replicate), and percent lysosomes positive for intraluminal vesicles (right; n>10 cells per replicate) from at least three independent biological replicates

are shown, represented by individual data points; error bars show SD. *p=0.0337, **p=0.0082 (one-way Anova followed by Dunnett's test).

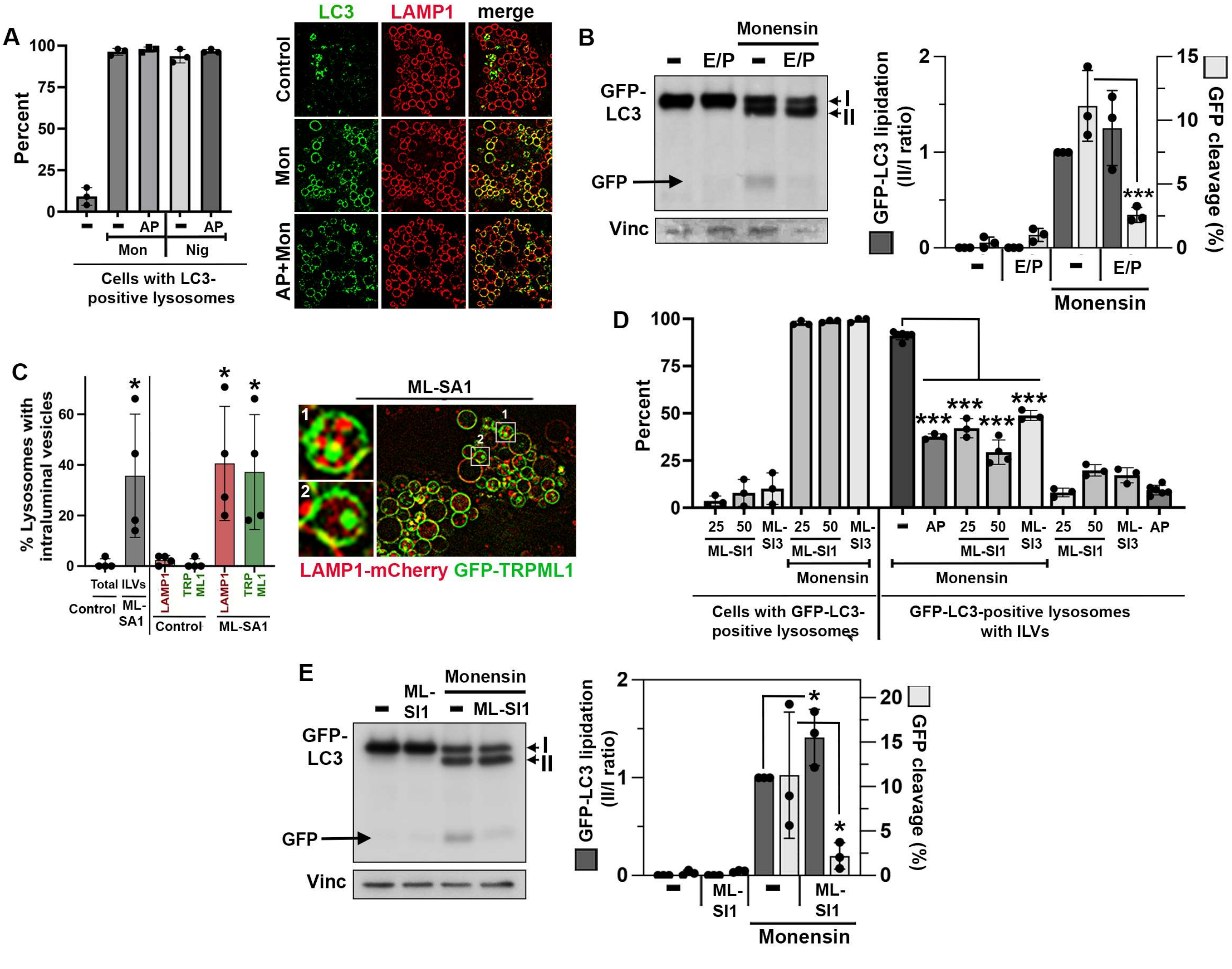

Supplementary Figure 2

Supplementary Figure 2: TRPML1 mediates ILV formation downstream of PIKfyve. (A) Images show cells expressing GFP-LC3 (green) incubated with membrane dye FM4-64 (red) and treated with Monensin to induce microautophagy. Graphs show quantification of GFP-LC3 and FM4-64-positive intraluminal vesicle populations through live imaging analysis. Percent lysosomes positive for intraluminal vesicles (n>10 cells per replicate) from at least three independent biological replicates are shown, represented by individual data points; error

bars show SD; ***p=0.0005; one-way Anova followed by Dunnett's test.) Images show immunofluorescence staining of endogenous LC3 (green) and LAMP1 (red) in cells treated with Monensin, or AP and Monensin. Graph depicts percent cells with five or greater LC3-positive lysosomes from three independent biological replicates, with n=100 cells per replicate for each condition. Error bars show SD. (B) Western blot shows GFP-LC3 lipidation and GFP cleavage in response to treatment with Monensin in the presence or absence of E64d and pepstatin A in *sgATG13* cells. Percent free GFP was compared between the indicated conditions using a one-way Anova followed by Dunnett's test (***p=0.0002). (C) Imaging shows cells expressing LAMP1-mCherry (red) and GFP-TRPML1 (green) treated with the TRPML1 activator ML-SA1 to induce microautophagy. Graph shows percent lysosomes with ILVs (n>10 cells). p-values from left to right: *p=0.0295, *p=0.0149, *p=0.0191 (Unpaired t test). (D) Graphs show quantification of GFP-LC3 lipidation and GFP-LC3-positive intraluminal vesicle populations through live imaging analysis. Percent cells positive for GFP-LC3 labeled lysosomes (left; n=100 cells per replicate), and percent lysosomes positive for intraluminal vesicles (right; n>10 cells per replicate). AP, ML-SI1 and ML-SA3 significantly inhibit the presence of ILVs in Monensin-treated cells (***p<0.0001; one-way Anova followed by Dunnett's test). (E) Representative Western blot shows GFP-LC3 lipidation and GFP cleavage in response to treatment with Monensin in the presence or absence of the TRPML1 inhibitor ML-SI1 in *sgATG13* cells. Percent free GFP and the GFP-LC3-II/I ratio in Monensin-treated cells were compared to cells treated with Monensin and ML-SI1 using a one-way Anova followed by Dunnett's test (from left to right; *p=0.0201, *p=0.0382).
